# Latent density discrepancies in commercial lung‐equivalent inserts and their clinical dosimetric impact

**DOI:** 10.1002/acm2.70668

**Published:** 2026-06-25

**Authors:** Minoru Nakao, Shuichi Ozawa, Hideharu Miura, Masahiro Hayata, Kosaku Habara, Masahiro Kenjo

**Affiliations:** ^1^ Hiroshima High‐Precision Radiotherapy Cancer Center Hiroshima Japan; ^2^ Department of Radiation Oncology, Graduate School of Biomedical & Health Sciences Hiroshima University Hiroshima Japan

**Keywords:** CT number calibration, quality assurance, treatment planning

## Abstract

**Background:**

Accurate heterogeneity correction in high‐precision radiotherapy relies on precise computed tomography (CT) number‐to‐density conversion via the Hounsfield unit look‐up table (HLUT). While physical properties of tissue‐equivalent materials are generally assumed consistent with manufacturer specifications, an independent audit identified clinically significant density discrepancies in commercially available lung‐equivalent phantom inserts.

**Purpose:**

This study evaluates the physical properties of nonconforming lung inserts through mass measurements and stoichiometric analysis, and assesses the clinical dosimetric impact of the associated density discrepancies.

**Methods:**

Five lung‐inhale inserts manufactured in 2010, 2015, and 2024 (10A, 10B, 15A, 15B, and 24A) were analyzed. Mass and physical dimensions were measured in triplicate using a precision balance (1 mg resolution) and vernier calipers. Stoichiometric analysis was conducted using reference materials to evaluate the tissue‐equivalence of the inserts and quantify deviations from the theoretical baseline. A nonconforming table and a conforming reference table (RT) were established, derived from inserts 10A and 24A, respectively. For clinical impact assessment, volumetric modulated arc therapy (VMAT) plans for three clinical cases involving centrally located lung tumors (utilizing both inspiration breath‐hold (IBH) and free‐breathing) were optimized for stereotactic body radiotherapy (SBRT) and recalculated with the RT using the Acuros XB algorithm. Differences in gross tumor volume (GTV) mean dose and planning target volume (PTV) D95% were evaluated to quantify the dosimetric consequences.

**Results:**

The 2010 inserts (10A and 10B) exhibited a 17.1% mass reduction and lower CT numbers compared to the reference 24A insert. Dimensional variations were negligible (≤ 0.2 mm) across all samples. Clinical recalculation revealed maximum dose reductions of 2.1% for the GTV mean dose and 3.0% for the PTV D95% in the worst‐case scenario. These errors exceed the 2% clinical tolerance, propagated by HLUT interpolation across the low‐density range.

**Conclusions:**

Substantial inter‐lot density variations in commercial calibration phantoms can lead to dosimetric errors that exceed established clinical limits, particularly for centrally located tumors treated with IBH. Medical physicists must not implicitly rely on nominal manufacturer values; independent audits and initial mass screening at acceptance are highly recommended for maintaining dose calculation accuracy.

## 1 INTRODUCTION

Accurate dose calculation is fundamental for achieving high‐precision dose distributions in radiotherapy, particularly in stereotactic body radiotherapy (SBRT)^1^, ^2^, ^3^ for lung cancer, where steep dose gradients and tissue heterogeneities are present. Due to the low physical density of lung tissue, small absolute errors in computed tomography (CT) number‐to‐density (CT‐to‐density) conversion produce significant errors in dose calculation, particularly for heterogeneity correction algorithms such as Acuros XB (Varian Medical Systems, Palo Alto, CA, USA). Although American Association of Physicists in Medicine (AAPM) Report No. 85 recommends that heterogeneity correction accuracy be within 2% of the prescribed dose for megavoltage photon beams, latent density variations in commercial phantom materials can cause the calculated dose error to exceed this clinical tolerance.^4^

Treatment planning systems (TPSs) rely on a CT‐to‐density conversion table, referred to as a Hounsfield unit look‐up table (HLUT), to assign electron or physical densities to patient anatomy based on CT numbers. To verify the reliability of the registered HLUT, the CT number calibration audit (CTCA)^5^, ^6^, ^7^ has been developed and implemented. Prior multi‐institutional audits and quality assurance (QA) programs have revealed that phantom material variability can be a significant source of dosimetric error. For instance, discrepancies between the nominal and actual physical densities of commercial water‐equivalent phantoms have been shown to introduce systematic absolute dose calibration errors.^8^ Furthermore, investigations into low‐density lung‐equivalent materials for the Imaging and Radiation Oncology Core QA program have demonstrated significant sample‐to‐sample density variability, emphasizing that such materials must be independently validated with each new batch to prevent dosimetric errors.^9^ While these previous audits have focused on user‐generated errors in HLUT construction or CT scanner instability, no study has quantified the dosimetric impact of latent density discrepancies originating from the manufacturer's own lot‐to‐lot variability in lung‐equivalent inserts.

In clinical practice, the physical properties of commercially available tissue‐equivalent inserts are generally assumed to remain stable and consistent with manufacturer specifications. However, during a CTCA, a lung‐equivalent insert was found to have a CT number deviation exceeding the established tolerance levels.^10^, ^11^ In the CTCA protocol, tolerance levels are defined based on density discrepancies that can cause dose differences exceeding 2%, and inserts exceeding these levels are classified as nonconforming. This nonconforming insert exhibited a lower density than specified, which introduced systematic errors into the clinical HLUT.

The discovery of this error prompted a comprehensive evaluation to quantify its dosimetric impact on lung SBRT. In this study, we investigate the clinical consequences of using an HLUT derived from such a nonconforming insert by comparing the resulting dose distributions with those generated from a conforming table. By demonstrating the potential risks associated with these discrepancies, we emphasize the role of the CTCA in identifying material‐related uncertainties that could compromise the accuracy of high‐precision radiotherapy.

## 2 METHODS

### 2.1 Lung‐equivalent inserts

The investigation focused on lung (inhale) inserts manufactured by CIRS (Sun Nuclear, Norfolk, VA, USA). Two specific inserts manufactured in 2010 (referred to as Lung10A and Lung10B) were identified as exhibiting significant deviations during the audit. Three additional inserts were included in the analysis for comparison: two manufactured in 2015 (Lung15A and Lung15B) obtained from another institution, and one manufactured in 2024 (Lung24A).

To verify geometric consistency, the diameter and length of all five inserts were measured in triplicate using vernier calipers. Dimensional variations were confirmed to be within 0.2 mm across all samples. A thin labeled disk was attached to the end of each insert, indicating its nominal physical and electron densities. As the nonremovable disks precluded direct measurement of the insert density, we instead compared their total mass to evaluate any discrepancies. The total mass of each insert was measured using a precision balance with a resolution of 1 mg. Given the consistent volumes, mass measurements were served as a surrogate for density evaluation.

### 2.2 Stoichiometric analysis

The CTCA protocol was used to independently evaluate the HLUTs. This method generates a reference theoretical HLUT using the stoichiometric CT number calibration approach based on two reference materials with independently determined elemental compositions and densities: Tough Lung and Tough Bone (Kyoto Kagaku, Kyoto, Japan). Unlike standard commercial inserts, the precise chemical compositions and physical densities of these materials were determined through independent elemental analysis, following validated methodologies established in our previous studies.^12^

The primary focus of this study was to verify the physical density of the lung (inhale) inserts. The five test lung inserts were scanned simultaneously with the reference materials using a GE Optima 580 CT scanner (GE Medical Systems, Waukesha, WI, USA). The scanning parameters were set to a tube voltage of 120 kVp, a slice thickness of 2.5 mm, a standard reconstruction kernel, and a scan field of view of 500 mm. To minimize CT artifacts caused by the high density of the materials, the phantoms were positioned with sufficient separation.

A theoretical HLUT was generated using the stoichiometric method based on the measured CT numbers of the reference materials. This HLUT served as the baseline for converting the measured CT numbers of the five test lung inserts into their corresponding theoretical physical densities. Finally, these derived densities were compared against the nominal physical densities indicated on each insert's labeled disk to evaluate the magnitude of any discrepancies.

### 2.3 Dosimetric impact assessment using clinical cases

To evaluate the impact of the density deviations on patient dose distributions, we performed a retrospective dosimetric analysis for three patients with lung cancer located in the central lung region. The cases included patients irradiated during either inspiration breath‐hold (IBH) or free breathing (FB). This analysis was conducted under ethical approval granted by the institutional review board of the author's institution (Approval No. E2017‐0947‐02).

Two distinct HLUTs were generated based on the measured data of the Lung10A (nonconforming table, NCT) and the baseline Lung24A (reference table, (RT)) inserts, respectively. These tables were registered in the TPS, Eclipse (version 16, Varian Medical Systems, Palo Alto, CA, USA). The CT images for treatment planning were acquired under either FB or IBH conditions. IBH was utilized to expand the lung volume and minimize the dose to mediastinal structures, including the heart, while FB was employed for cases where respiratory tumor motion was confirmed to be minimal.

**FIGURE 1:**
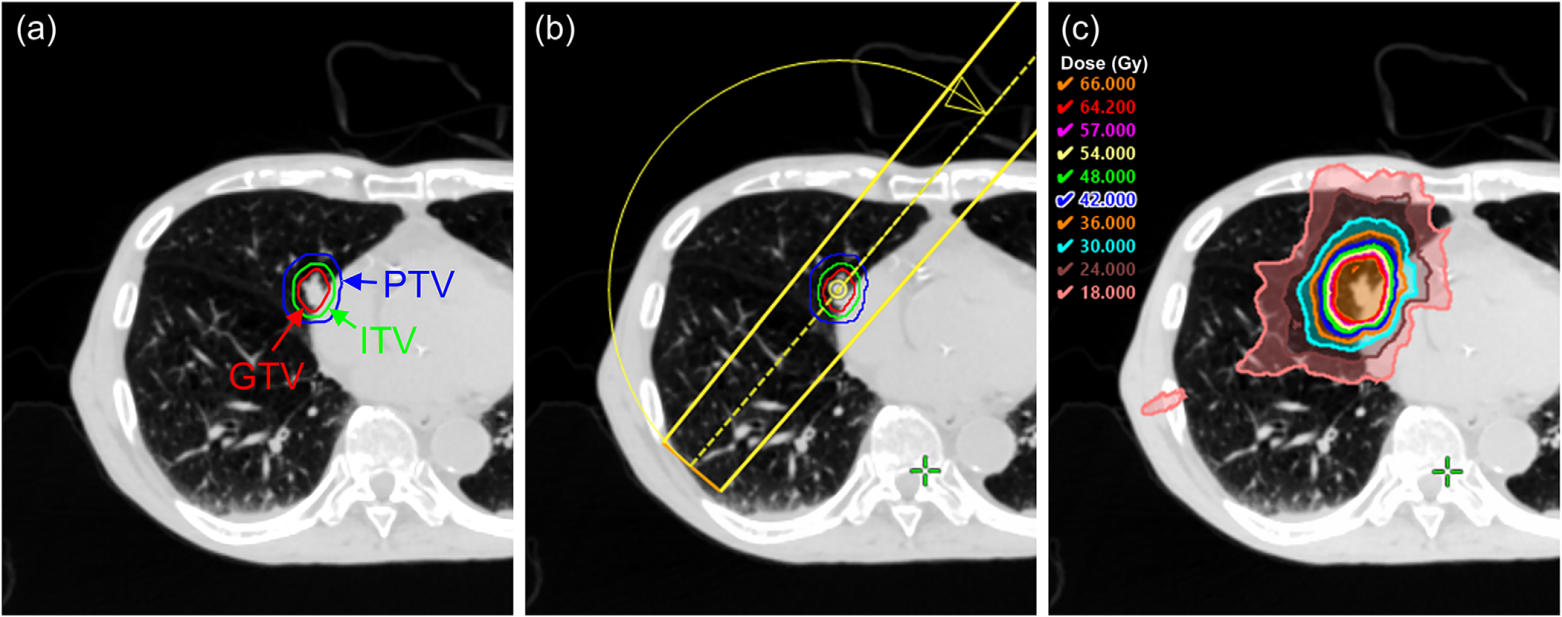
Overview of a representative clinical case (Patient Case 1) used for the dosimetric impact assessment. (a) Axial CT image showing the delineation of the GTV, ITV, and PTV. (b) Beam arrangement for the VMAT plan utilizing a partial arc. (c) Resulting dose distribution.

Initial treatment plans for each case were optimized using the NCT. Dose distributions were calculated using the Acuros XB algorithm (version 16.1.2) in dose‐to‐medium mode. Each plan was designed for a TrueBeam STx linear accelerator (Varian Medical Systems) using a 6 MV flattening filter‐free beam. The volumetric modulated arc therapy (VMAT) plans were normalized such that a prescription dose of 60 Gy in 8 fractions was assigned to the 80% isodose level, ensuring that 95% of the planning target volume (PTV) was covered by the prescription dose. This normalization strategy resulted in a dose distribution where the maximum dose reached approximately 125% of the prescription dose, with high‐dose regions intentionally concentrated within the gross tumor volume (GTV). Figure 1 illustrates a representative example from Case 1, showing the contouring of the GTV, internal target volume (ITV), and PTV, along with the beam arrangement and dose distribution.

Subsequently, the dose distributions were recalculated using the RT while maintaining the monitor units and beam parameters identical to those of the initial NCT‐based plans. To quantify the dosimetric impact, the following metrics were evaluated: mean dose to the GTV; *D*_98%_ and *D*_95%_ of the PTV, in which *D*_X%_ was the dose received by X% of the PTV volume; *V*_100%_ of the PTV, in which *V*_100%_ was the PTV volume receiving at least 100% of the prescription dose; and the Paddick conformity index (CI).^13^ The CI was calculated as follows:

(1) $$\mathrm{CI}=\frac{V_{\mathrm{PTV}\left(100\%\right)}^{2}}{V_{\mathrm{PTV}}\times V_{100\%}}$$

where $V_{\mathrm{PTV}(100\%)}$ is the volume of the PTV receiving the prescribed dose, $V_{\mathrm{PTV}}$ is the total volume of the PTV.

Additionally, 3D gamma analysis was performed to evaluate the spatial dose discrepancies using an in‐house program based on the algorithm of Low et al.^14^ The analysis utilized global normalization relative to the prescription dose (60 Gy), with a 10% dose threshold applied to exclude low‐dose background noise, consistent with AAPM Task Group 218.^15^ The gamma pass rates were calculated using two sets of criteria: 2%/2 mm and 2%/1 mm.

### 2.4 Generative AI for manuscript preparation

The preparation of this manuscript was assisted by a generative artificial intelligence (AI) tool, Gemini 3 Pro (Google LLC, Mountain View, CA, USA), to enhance linguistic accuracy and clarity. The authors carefully reviewed and revised all AI‐generated suggestions and assume full responsibility for the final content.

## 3 RESULTS

### 3.1 Physical properties and CT numbers of the lung inserts

All five lung inserts were visually indistinguishable in terms of color and external appearance. Table 1 summarizes the physical characteristics of the inserts, including year of manufacture, nominal density, measured mass, measured CT number, and calculated theoretical density.

**TABLE 1:** Comparison of physical properties and CT numbers (Mean ± SD) for the five lung‐equivalent inserts.

Name	Year	Nominal density (g/cm^3^)	Mass (g)	CT number (HU)	Theoretical density (g/cm^3^)
Lung10A	2010	0.2	10.05	−849 ± 1	0.141
Lung10B	2010	0.2	10.13	−840 ± 2	0.150
Lung15A	2015	0.2	12.07	−798 ± 1	0.191
Lung15B	2015	0.2	12.03	−786 ± 1	0.203
Lung24A	2024	0.205	12.12	−787 ± 1	0.203

**Abbreviations**: CT, computed tomography; SD, standard deviation.

The measured mass represents the average of three repeated measurements, with a standard deviation (SD) of less than 3 mg. CT numbers are reported as the mean ± SD obtained from the region of interest across three scans. The theoretical densities of the five test inserts were derived from their measured CT numbers using the baseline HLUT. This HLUT was constructed by applying the stoichiometric method to the Tough Lung and Tough Bone reference materials (as described in Section 2.2).

Substantial discrepancies were observed between the specific inserts tested from the 2010, 2015, and 2024 manufacturing batches. Although the nominal density was labeled as approximately 0.2 g/cm^3^ for all inserts, the measured mass of the 2010 inserts was significantly lower than that of the newer lots. Specifically, the mass of Lung10A was 17.1% lower than that of the baseline Lung24A (10.05 g vs. 12.12 g). Correspondingly, the CT numbers for the 2010 inserts (Lung10A and Lung10B) were notably lower (−849 HU and −840 HU, respectively) compared to the newer (2015 and 2024) inserts, which ranged from −798 HU to −786 HU. Consequently, the calculated theoretical densities for the specific 2010 inserts (0.141–0.150 g/cm^3^) showed a marked deviation from their nominal values, whereas the 2015 and 2024 lots were consistent with the manufacturer's specifications.

### 3.2 Stoichiometric analysis

**FIGURE 2:**
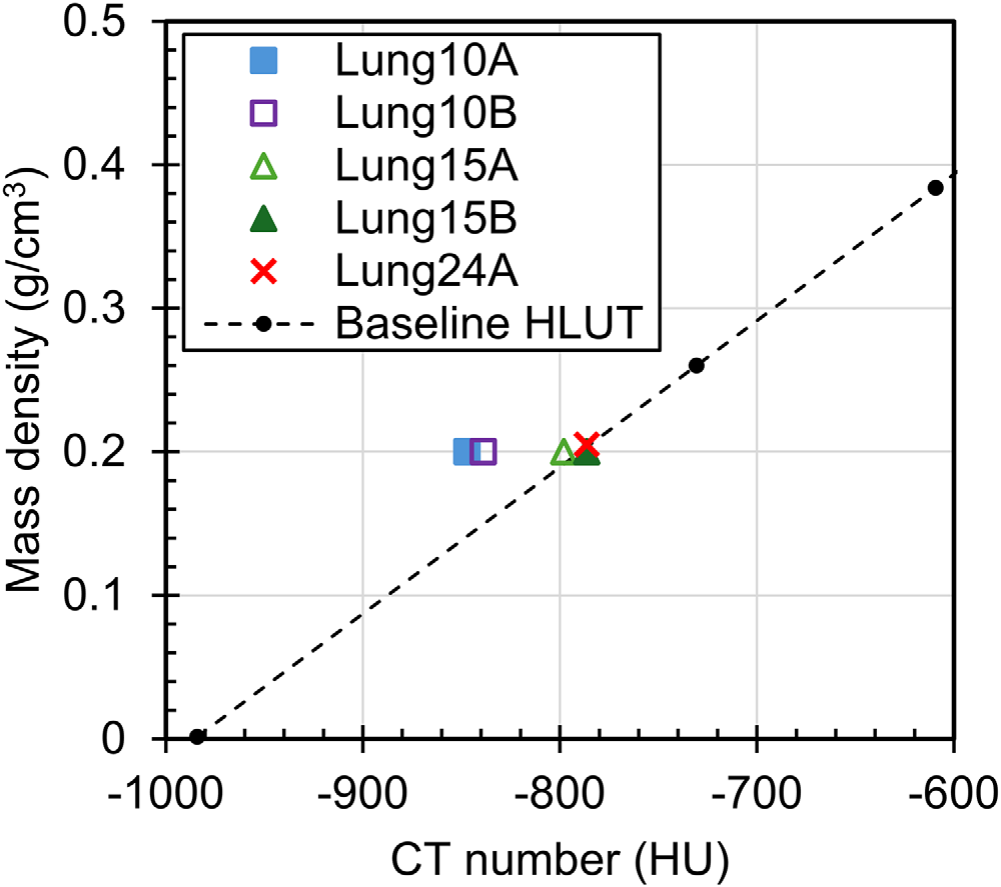
Comparison between the measured data of the five CIRS lung (inhale) inserts and the baseline HLUT in the lung density region.

Figure 2 illustrates the comparison between the measured CT numbers and nominal densities for the five lung inserts relative to the baseline HLUT. The inserts from the 2015 and 2024 lots (Lung15A, Lung15B, and Lung24A) closely conformed to the theoretical calibration curve, indicating that their physical properties were consistent with the stoichiometric method. In contrast, the 2010 inserts (Lung10A and Lung10B) exhibited marked deviations from the theoretical curve, producing notably lower CT numbers than expected for their nominal physical density of 0.2 g/cm^3^.

The theoretical physical densities calculated using the baseline HLUT are summarized in Table 1. The differences between the nominal and theoretical physical densities were most pronounced for the 2010 lot, with differences of 0.059 g/cm^3^ for Lung10A and 0.050 g/cm^3^ for Lung10B. For the newer lots (2015 and 2024), the differences were negligible, ranging from −0.003 to 0.009 g/cm^3^.

### 3.3 Dosimetric impact in the clinical case

**FIGURE 3:**
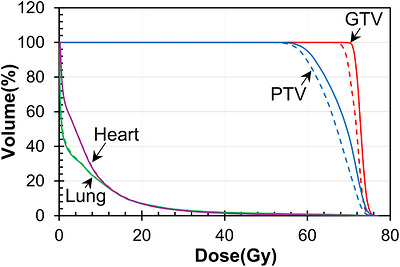
DVHs for Case 1 comparing the plans calculated with the NCT (solid lines) and the RT (dashed lines).

Figure 3 illustrates the dose‐volume histograms (DVHs) for Case 1, which represents the worst‐case scenario in terms of target coverage degradation, comparing the initial plan optimized using the NCT (derived from Lung10A) and the recalculated dose distribution using the RT (derived from Lung24A). Table 2 summarizes the characteristics of each clinical case and the corresponding dosimetric impacts. Across all evaluated cases, recalculation with the RT revealed a systematic reduction in target dose levels and coverage metrics.

**TABLE 2:** Summary of dosimetric parameters and gamma pass rates for lung SBRT cases with a centrally located lung tumor.

Patient	Respiratory Control	GTV Mean (Gy) NCT / RT	Volume (cm^3^) PTV / Lung*	PTV *D*_98%_ (Gy) NCT / RT	PTV *D*_95%_ (Gy) NCT / RT	PTV *V*_100%_ (%) NCT / RT	PTV CI NCT / RT	Gamma pass rate (%) 2%/2 mm / 2%/1 mm
Case 1	IBH	72.9 / 71.6	20.7 / 13.3	58.2 / 56.5	60 / 58.2	95.0 / 89.3	0.936 / 0.872	99.3% / 96.9%
Case 2	IBH	75.6 / 75.6	23.6 / 15.3	58.2 / 57.2	60 / 59.1	95.0 / 92.8	0.876 / 0.862	100.0% / 99.3%
Case 3	FB	71.7 / 71.2	73.7 / 39.8	58.7 / 57.3	60 / 58.7	95.0 / 90.3	0.849 / 0.890	99.5% / 97.5%
Mean	–	73.4 / 72.8	39.3 / 22.8	58.4 / 57.0	60 / 58.7	95.0 / 90.8	0.887 / 0.874	99.6% / 97.9%

**Abbreviations**: CI, Paddick conformity index; FB, free breathing; GTV, gross tumor volume; IBH, inspiration breath‐hold; Lung*, volume of lung tissue within the PTV; NCT, nonconforming table; PTV, planning target volume; RT, reference table; SBRT, stereotactic body radiotherapy.

In Case 1 (the worst‐case), the mean dose to the GTV was 72.9 Gy with the NCT, whereas it decreased to 71.6 Gy upon recalculation with the RT. Similarly, the *D*_95%_ of the PTV decreased from 60.0 Gy to 58.2 Gy. When calculated relative to the prescription dose of 60 Gy, these dose reductions in Case 1 amounted to a 2.1% decrease in the GTV mean dose and a 3.0% decrease in the PTV *D*_95%_. Overall, while the plans were initially normalized to PTV *V*_100%_ = 95.0% using the NCT, the mean *V*_100%_ across all cases dropped to 90.8%. The most pronounced reduction in coverage was observed in Case 1, where the PTV *V*_100%_ dropped from 95.0% to 89.3%.

In contrast to the consistent reduction in target coverage, the Paddick CI exhibited divergent trends across the cases. In Cases 1 and 2, the CI showed a decrease, with Case 1 showing the most marked degradation from 0.936 to 0.872. Conversely, Case 3 showed an increase in CI from 0.849 to 0.890. Regardless of the CI trends, the dosimetric differences for organs at risk, specifically the ipsilateral lung and heart, remained minimal.

**FIGURE 4:**
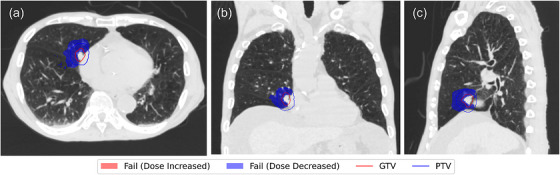
3D gamma analysis results for the worst‐case scenario (Case 1) evaluated with the 2%/1 mm criteria. Axial, coronal, and sagittal views are shown. Failing voxels (indicated in blue) represent regions where the dose recalculated with the RT was lower than the initial calculation.

Figure 4 presents the 3D gamma analysis results for the worst‐case scenario (Case 1). The spatial dose discrepancies resulted in gamma pass rates of 99.3% for the 2%/2 mm criteria and 96.9% for the 2%/1 mm criteria (Table 2). As shown in the axial, coronal, and sagittal views, the failing voxels identify regions where the dose recalculated with the RT was lower than the initial calculation. These discrepancies were primarily concentrated at the periphery of the target volume and within the low‐density lung tissue, where the overestimation of density in the NCT had the most prominent impact on the dose calculation algorithm.

## 4 DISCUSSION

This study highlights the presence of latent errors in the HLUT that may remain undetected for years, emphasizing that such discrepancies translate into clinically non‐negligible dosimetric errors. Our findings, identified through an independent third‐party audit, demonstrate that a systematic shift in CT‐to‐density conversion can compromise the accuracy of high‐precision radiotherapy.

The nonconforming inserts (Lung10A and Lung10B) were manufactured in 2010. Although their average CT numbers were registered in the TPS as recently as 2022, the density error was only revealed during the 2024 audit. A critical observation in this study was the dimensional stability of the inserts; variations in diameter and length were within 0.2 mm (less than 0.7% of the diameter) across all samples. This confirms that the physical volume remained nearly identical to the nominal specifications despite the passage of over a decade. Therefore, the lower average CT numbers and measured mass indicate an inherent reduction in material density rather than physical degradation such as swelling or shrinkage. While the root cause, which could be lot‐to‐lot manufacturing variability or long‐term temporal degradation of the lung‐equivalent foam, falls outside the scope of this work, the essential lesson for medical physicists is clear. Nominal manufacturer values should not be accepted unequivocally without independent verification. Although the exact prevalence of such material‐level defects across radiotherapy centers remains unknown, their potential clinical impact is high. Unlike common registration errors,^6^ these intrinsic material non‐conformities are virtually impossible to detect through visual inspection or routine commissioning, necessitating independent verification.

We focused our clinical impact assessment on Case 1 to define the “risk ceiling,” as this centrally located tumor treated with the IBH technique represents a worst‐case scenario. Recalculation with the RT revealed dose reductions of 2.1% for the GTV mean dose and 3.0% for the PTV *D*_95%_. Given that the established tolerance for heterogeneity correction algorithms is 2%,^4^ these results demonstrate that errors introduced by the NCT can exceed clinical limits. This threshold is further reinforced by recent work from Fogazzi et al.,^16^ who underscored that dose distributions must remain accurate within 1%–2% to maintain the overall clinical uncertainty budget. Consequently, the 3% systematic error identified in this context represents an unacceptable deviation from the critical benchmarks required for modern high‐precision radiotherapy. Regarding the conformity metrics, while Cases 1 and 2 showed a predictable decrease in the CI, Case 3 exhibited a paradoxical increase (from 0.849 to 0.890), which is attributed to its large PTV volume (73.7 cm^3^). Upon dose recalculation using the RT, the ratio of $V_{\mathrm{PTV}(100\%)}$ (RT plan relative to NCT plan) was maintained at 0.99, whereas the ratio of the total prescription volume (*V*_100%_) decreased to 0.94. Because the reduction in the denominator (*V*_100%_) outpaced that of the squared numerator in Equation 1 (0.99^2^ = 0.98), the CI for Case 3 was mathematically improved.

Furthermore, the impact of such density discrepancies is likely amplified by the choice of dose calculation algorithm. Previous studies have indicated that Acuros XB is more sensitive to CT number variations in low‐density regions compared to the Analytical Anisotropic Algorithm.^17^ Because the HLUT in the lung density range is often constructed via interpolation between a limited number of calibration points, such as lung‐inhale and lung‐exhale, a deviation in even a single insert may propagate systematic biases across the entire low‐density range. This creates a hidden systematic error that is notoriously difficult to isolate through standard end‐to‐end testing. As noted by Edwards et al., the complexity of anthropomorphic phantoms often masks specific dose calculation errors among other failure modes, such as setup or delivery uncertainties.^18^ This hidden risk is further demonstrated by our 3D gamma results, where overall pass rates remained high (> 96% for 2%/2 mm and 2%/1 mm) despite a 3.0% decrease in the PTV *D*_95%_. This discrepancy stems from the steep dose gradients of SBRT and the mechanics of global gamma evaluation, where the analyzed low‐to‐mid dose volume (≥ 6 Gy) is much larger compared to the PTV. Under global normalization based on the prescribed dose (1.2 Gy tolerance for 2%), the large number of passing voxels outside the target minimizes the relative impact of the failing voxels inside the PTV, masking the localized clinical underdosage.

Ultimately, since the goal of commissioning is to establish a safe and high‐precision environment for all patients, rigorous verification of phantom insert quality during acceptance testing is imperative. While mass measurement serves only as a simple screening tool to notice potential lot‐to‐lot variations, the density evaluated from measured mass and volume remains a rough estimate due to the presence of nonremovable disks. Therefore, the clinical utility of independent third‐party audits remains paramount for accurately identifying and quantifying latent material errors that routine protocols might overlook.

As an alternative to single‐energy CT (SECT), dual‐energy CT (DECT) approaches are under continuous investigation due to their potential to solve material ambiguities. Unfortunately, despite intense research in DECT, dedicated scanners equipped with this modality are still not available for scanning patients in most radiation therapy centers.^16^ Thus, SECT remains the standard imaging modality applied for radiation treatment planning, and our methodology provides a practical tool for verifying HLUT integrity, readily accessible to the majority of clinics relying on standard SECT.

For institutions that cannot readily access external audit services, stoichiometric analysis offers a practical alternative for identifying material discrepancies. By integrating validated algorithms from public sources, such as the stoichiometric calibration tools provided in the European Particle Therapy Network (EPTN) GitHub repository,^19^ medical physicists can effectively verify the integrity of their HLUT using a standardized framework circumventing the necessity of developing complex programs from scratch. In clinical practice, when an out‐of‐tolerance insert is identified, applying a theoretical HLUT provides an immediate solution to restore accurate dose calculations.

However, replacing measured calibration points with a theoretical HLUT must be approached with caution. For example, our method utilizes reference plugs (Tough Lung and Tough Bone) whose elemental compositions were independently analyzed^12^ and validated through multi‐institutional studies,^6^ while the EPTN protocol rigorously verifies tissue equivalence via proton range measurements.^20^ If a significant discrepancy is identified without such verified reference baselines, it should serve as a trigger to consult the manufacturer regarding the insert's quality. Furthermore, although this study captured inter‐lot variability rather than longitudinal degradation, routine CT scanning of phantom inserts to accumulate long‐term CT number trend data is highly recommended. Monitoring these trends will allow clinics to detect temporal degradation, establishing a clear baseline for when to consult the manufacturer regarding replacement. Another limitation of this study is the small sample size per manufacturing batch, as we analyzed only two inserts from 2010, two from 2015, and one from 2024. Therefore, because only one or two inserts per batch were available, these findings should be considered indicative rather than definitive for entire production lots, and within‐lot variability remains unquantified.

## 5 CONCLUSIONS

This study demonstrated that discrepancies between nominal and evaluated densities may arise even in commercially available tissue‐equivalent materials. Such inter‐lot variability presents a latent risk of dosimetric errors that may exceed clinical tolerances, leading to dose reductions of up to 3.0% for target coverage in specific clinical scenarios, such as centrally located lung tumors. Because identifying these discrepancies is challenging through standard institutional QA procedures alone, independent third‐party audits serve as a vital tool for verifying HLUT accuracy. Utilizing such external audits, complemented by simple mass screening at acceptance, is highly recommended for maintaining dose calculation accuracy.

## AUTHOR CONTRIBUTIONS

**Minoru Nakao**: Conceptualization; methodology; writing—original draft. **Shuichi Ozawa**: Formal analysis; investigation; writing—review & editing. **Hideharu Miura**: Formal analysis; investigation; writing—review & editing. **Masahiro Hayata**: Formal analysis; investigation; writing—review & editing. **Kosaku Habara**: Formal analysis; investigation; writing—review & editing. **Masahiro Kenjo**: Formal analysis; resources; writing—review & editing.

## CONFLICT OF INTEREST STATEMENT

The authors have no relevant conflicts of interest to disclose.

## ETHICAL APPROVAL

This study was approved by the Hiroshima University Institutional Review Board (approval no. E2017‐0947‐02).

## Data Availability

The data that support the findings of this study are available from the corresponding author upon reasonable request.
